# Influence of growth hormone treatment on radiographic indices of the spine: propensity-matched analysis

**DOI:** 10.1186/s13018-017-0630-z

**Published:** 2017-09-06

**Authors:** Yeo-Hon Yun, Soon-Sun Kwon, Youngdo Koh, Dong-Jun Kim, Jonghyun Ahn, Seung Yeol Lee

**Affiliations:** 1 Department of Orthopaedic Surgery, Ewha Womans University Mokdong Hospital, 1071 Anyangcheon-ro, Yangcheon-gu, Seoul, 07985 Korea; 2 Department of Mathematics, College of Natural Science, Ajou University, Gyeonggi, Korea

**Keywords:** Growth hormone, Scoliosis, Propensity-matched analysis

## Abstract

**Background:**

We performed this study to investigate the influence of recombinant human growth hormone (rhGH) therapy on radiographic indices of the spine using propensity-matched analysis.

**Methods:**

Patients with idiopathic short stature who had undergone both growth hormone therapy and whole-spine radiographs more than twice prior to 15 years of age were included in the patient group. Other patients who had undergone whole-spine radiographs more than twice prior to the same age during regular checkups for idiopathic scoliosis formed the control group. Propensity-matched analysis was performed to reduce the selection bias. The scoliosis Cobb angle, coronal balance, apical vertebral translation, apical rotation, and pelvic obliquity were measured from the radiographs taken at the periodic follow-ups. The rate of progression of the measurements was adjusted by multiple factors using a linear mixed model with sex as the fixed effect and age and each subject as the random effects.

**Results:**

Using a propensity-matched analysis, 48 patients were finally included in both groups. The scoliosis Cobb angle increased by 1.0° (*p* < 0.001) per year in the patient group, whereas there was no significant annual change in the control group (*p* = 0.496). Female patients showed a greater scoliosis Cobb angle (1.8°, *p* = 0.039) compared with male patients. There was no significant difference between the patient and control groups in coronal balance (*p* = 0.264). Apical vertebral translation per year was increased by 1.2 mm (*p* < 0.001) in the patient group and 0.5 mm in the control group (*p* = 0.003).

**Conclusion:**

Radiographic examination revealed that growth hormone therapy for idiopathic short stature affected the progression of the scoliosis Cobb angle and apical vertebral translation on the coronal plane. Physicians should be aware that annual follow-up is required to evaluate the change in the curvature of the spine in patients undergoing rhGH treatment.

## Background

Purified human growth hormone (hGH) [1] has been used in patients with pituitary dwarfism for over 50 years [2] and for other conditions that cause dwarfism. More recently, recombinant hGH (rhGH) has replaced hGH in the treatment of dwarfism instead of hGH, as the latter poses a risk of transmitting Creutzfeldt-Jakob disease [3]. GH replacement increases bone, fat, and muscle mass and results in sustained improvement in the quality of life for the recipient [4].

However, rhGH replacement therapy is associated with several adverse effects in children including prepubertal gynecomastia [5] and malignancy [6, 7]. Regarding musculoskeletal system effects, slipped capital femoral epiphysis in children receiving rhGH is reportedly more frequent than in the general population [8]. Scoliosis is also a major concern.

Whether or not GH therapy can actually cause the progression of scoliosis is debatable. A case of progression of structural scoliosis during treatment with GH was reported [9], and several other studies purported to show that GH therapy may exacerbate scoliosis [10, 11]. But, to the contrary, another study reported that the incidence of scoliosis in children treated with GH was about 4% [12], which was similar to that of idiopathic scoliosis in children in the general population [13].

Most of the studies of the adverse effects of GH therapy on the spine have been population-based prevalence and incidence studies [11, 12] or case series [9]. Data on the prevalence and incidence of scoliosis is not useful in determining the progression rate of the deformity. In a case series, a comparison of the patients with members of a control group who show similar growth rates should be performed to fairly access the effect of GH therapy alone on the progression of scoliosis, because the progression of scoliosis is influenced by the growth rate. Therefore, we performed this study to investigate the influence of rhGH therapy on radiographic indices of the spine using propensity-matched analysis.

## Methods

This study was approved by the institutional board of Ewha Womans University Mokdong Hospital (IRB number: 2015-02-016-004), which waived informed consent because of its retrospective nature.

We reviewed the medical records of consecutive patients who underwent whole-spine anteroposterior (AP) radiographs more than twice when they were ≤ 15 years of age, between March 2001 and February 2015. Patients who underwent GH therapy as a treatment of idiopathic short stature were included in the patient group. The subjects from which spine radiographs were taken for purposes of regular checkups for idiopathic adolescent scoliosis formed the control group. If surgery or bracing for scoliosis was performed during the follow-up, only the radiographs taken before the intervention were included. The exclusion criteria were diagnosis with neuromuscular disease or genetic disease, such as Turner syndrome, and inadequate radiographs available for review. Age at time of the examination, sex, type and duration of rhGH administration, and underlying disease were obtained by reviewing the medical records.

Whole-spine radiographs were obtained using a Digital Diagnost (Philips Research, Eindhoven, The Netherlands) with a source-to-image distance of approximately 180 cm and with the patient in the standing position. The radiograph setting depended on the patient’s body size. All radiograph images were digitally acquired using a picture archiving and communication system (STARPACS; Infinitt, Seoul, Korea), and measurements were subsequently carried out using PACS software.

### Measurement parameters

**Fig. 1:**
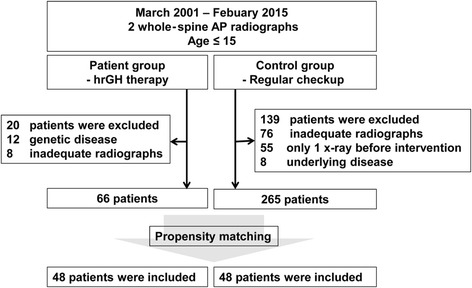
Inclusion and exclusion criteria

Five indices (scoliosis Cobb angle, coronal balance, apical vertebral translation, apical rotation, and pelvic obliquity) were measured on the AP radiographs of the spine [14]. The magnitude of scoliosis was quantified using the Cobb technique [15]. The scoliosis Cobb angle was measured on a major curve in the patients with double-curve scoliosis. Coronal balance was measured as the horizontal displacement of the centroid of C7 relative to the center sacral vertical line (CSVL) drawn perpendicular to the floor through the midline of the sacrum [16]. Apical vertebral translation, which reveals the regional balance of the spine, was the horizontal distance between the centroid of the apical vertebra and the CSVL [16]. If the apical vertebra was located at the thoracic spine, higher than T11, the lateral displacement of the apex of the coronal curve was measured from the C7 plumb line. The Nash-Moe scale was used to investigate apical vertebral rotation on AP radiographs [17]. Pelvic obliquity was defined as the angle between a horizontal line and a line drawn between the iliac crests (Fig. 1) [18].

### Propensity-matched analysis

In observational studies, there are often significant differences between the characteristic subjects of the treatment group (cases) and the no-treatment group (controls). These differences should be adjusted to reduce treatment selection bias and determine the treatment effect. Several methods can reduce this type of bias and, in effect, make the two groups more similar. Propensity score matching can reduce the selection bias in an observational study. The propensity scores represent the relationship between multiple characteristics and a status as the dependent variable. The score is the probability of receiving a case status. The single score was calculated using multivariate logistic regression, with the SAS Proc LOGISTIC procedure. A greedy algorithm was used to match individual patients who received treatment with patients who did not. The best matching was identical to the SAS greedy (5 → 1 digit) match Macro. This meant that the case group was first matched to the control group on five digits of the propensity score. For those that did not match, four-digit matches were identified, and this process continued down to a one-digit match of propensity scores.

### Constructing a linear mixed model

For the case and control groups, the AP radiographs of the spine (scoliosis Cobb angle, coronal balance, apical vertebral translation, apical rotation, and pelvic obliquity) were adjusted by multiple factors using a linear mixed model (LMM) with sex as the fixed effect and age and each subject as the random effects. The variance component covariance structure was used. Restricted maximum likelihood estimation was used to produce an unbiased estimator. By examining the individual pattern of the annual changes in radiographic indices of the spine through the follow-up duration, an LMM with a random slope and a random intercept was suggested. The adequacy of models was decided using the Akaike information criterion and the Bayesian information criterion. For model selection, a smaller value of each criterion is preferred. All models did have low scores and so were accepted as valid for representing the measurements.

### Statistical methods

Descriptive statistics including the mean and standard deviation were used to summarize patient demographics. The Kolmogorov-Smirnov test verified the normality of the distribution of variables. Data were analyzed using SAS version 9.4.2 (SAS Institute, Cary, NC). All statistics were two-tailed, and a *p* value < 0.05 was considered significant.

## Results

**Fig. 2:**
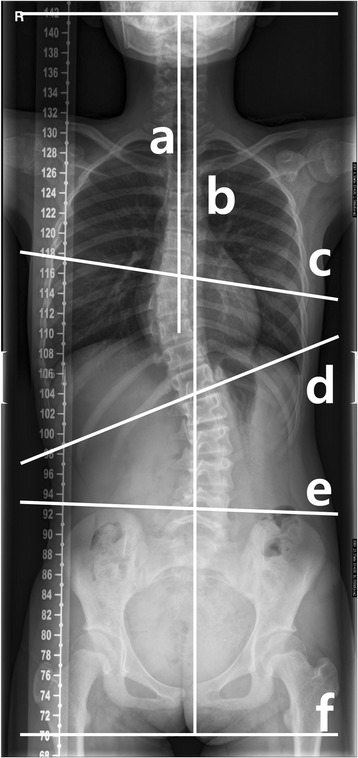
Radiographic measurements. The scoliosis Cobb angle was measured between c and d. Coronal balance was defined as the length between a and b. The angle of pelvic obliquity was measured between e and f. Apical vertebral translation was defined as the horizontal length between the center of apical vertebra and the C7 plump line. C7 plump line (**a**), center sacral vertical line (**b**), upper end plate of proximal end vertebra (**c**), lower end plate of distal end vertebra (**d**), line between both iliac crests (**e**), horizontal line (**f**)

In the patient group, a total of 86 patients who underwent rhGH therapy met the inclusion criteria. Following exclusions, 66 patients were included in the patient group of this study. In the control group, 404 subjects were screened and 265 patients met the inclusion criteria and were not eliminated by the exclusion criteria (Fig. 2). Using propensity-matched analysis, 48 patients were finally included in the control group in the present study (Fig. 2). Subject’s mean age at the time of examination was 11.8 ± 1.7 years (range, 8–15) in the patient group and 10.5 ± 3.5 years (range, 7–15) in the control group (Table 1). Of the patient group, 22 were treated with Saizen® and 26 were treated with Eutropin®, for idiopathic short stature. The mean dose of rhGH therapy was 0.21 ± 0.02 mg/kg/week (range, 0.15–0.25 mg/kg/week). The therapy was continued in all included patients during the follow-up period. The average number of whole-spine AP radiographs performed per patient was 3.2 (range, 2–6). The mean duration of follow-up was 16.5 ± 8.6 months (range, 6–35 months). Radiographic measurements are summarized in Table 1. There was no significant difference in the growth rates (*p* = 0.567) of the patient group (6.0 ± 0.4 cm per year) and control group (6.1 ± 0.4 cm per year).

**Table 1:** Patients’ demographics

	Screened data			After propensity matching		
	Patient group (*N* = 66)	Control group (*N* = 255)	*p* value	Patient group (*N* = 48)	Control group (*N* = 48)	*p* value
Age (years)	11.4 ± 2.1	12.7 ± 2.7	0.01	11.8 ± 1.7	10.5 ± 3.5	0.88
Sex (male/female)	38/28	96/196	< 0.001	26/22	22/26	0.90
Scoliosis Cobb angle (°)	8.5 ± 4.3	17.4 ± 9.8	< 0.001	8.9 ± 4.5	8.4 ± 5.0	0.59
Coronal balance (mm)	2.5 ± 9.0	4.2 ± 13.1	< 0.001	2.2 ± 8.2	3.2 ± 12.8	0.65
Apical vertebral translation (mm)	7.4 ± 5.3	14.9 ± 9.7	< 0.001	7.6 ± 5.7	7.9 ± 5.2	0.81
Apical rotation^a^	0.4 ± 0.5	0.4 ± 0.6	0.69	0.4 ± 0.5	0.3 ± 0.4	0.20
Pelvic obliquity (°)	1.5 ± 1.9	1.7 ± 2.0	< 0.001	1.6 ± 1.4	2.0 ± 2.3	0.34

^a^Nash-Moe scale

Scoliosis measured in the AP radiographs in patients who underwent rhGH therapy progressed to a greater extent than apparent in the control group radiographs as the patients grew older. In the patient group, the scoliosis Cobb angle increased by 1.0° (95% CI, 0.6–1.5°; *p* < 0.001) per year (Table 2). There was no significant annual change in the scoliosis Cobb angle in the control group (*p* = 0.496) (Table 3). Intergroup comparison also showed significant differences in the annual progression of the scoliosis Cobb angle (*p* < 0.001). Female subjects in the patient group showed a greater scoliosis Cobb angle (1.8°, *p* = 0.039) compared with male patients. Although coronal balance increased by 0.8 mm (95% CI, 0.2–1.5 mm; *p* = 0.012) in the control group, there was no significant difference between the patient and control groups with regard to coronal balance (*p* = 0.264). With regard to the regional valance, apical vertebral translation was increased by 1.2 mm per year (95% CI, 0.7–1.7 mm; *p* < 0.001) in the patient group and 0.5 mm (95% CI, 0.2–0.8 mm; *p* = 0.003) in the control group. The annual change of apical vertebral translation in the patient group was larger than that in the control group (*p* = 0.005). Apical vertebral rotation and pelvic obliquity did not progress in either group as subjects grew older (Tables 2 and 3).

**Table 2:** The estimation and fixed effects of radiographic indices using linear mixed models in the patient group

	Scoliosis Cobb angle			Coronal balance			Apical vertebral translation			Apical rotation			Pelvic obliquity		
	Estimation (95% CI)	SE	*p* value	Estimation (95% CI)	SE	*p* value	Estimation (95% CI)	SE	*p* value	Estimation (95% CI)	SE	*p* value	Estimation (95% CI)	SE	*p* value
Intercept	− 2.9	2.8	–	− 4.3	5.3	–	− 6.8	3.1	–	− 1.3	1.7	–	1.4	0.8	–
Age	1.0 (0.6, 1.5)	0.2	< 0.001	0.4 (− 0.5, 1.2)	0.4	0.42	1.2 (0.7, 1.7)	0.3	< 0.001	0.1 (− 0.2, 0.4)	0.1	0.48	0.0 (− 0.1, 0.1)	0.1	0.95
Sex	− 1.8 (− 3.4, − 0.1)	0.8	0.04	1.8 (− 1.4, 4.9)	1.6	0.27	− 1.8 (− 3.6, 0.1)	0.9	0.07	− 1.5 (− 2.5, − 0.4)	0.5	0.007	0.5 (− 0.1, 1.0)	0.3	0.08

*CI* confidence interval, *SE* standard error

**Table 3:** The estimation and fixed effects of radiographic indices using linear mixed models in the control group

	Scoliosis Cobb angle			Coronal balance			Apical vertebral translation			Apical rotation			Pelvic obliquity		
	Estimation (95% CI)	SE	*p* value	Estimation (95% CI)	SE	*p* value	Estimation (95% CI)	SE	*p* value	Estimation (95% CI)	SE	*p* value	Estimation (95% CI)	SE	*p* value
Intercept	11.1	2.0	–	− 10.6	3.9	–	3.0	1.9	–	− 1.0	1.3	–	2.3	0.7	–
Age	− 0.1 (− 0.4, 0.2)	0.2	0.50	0.8 (0.2, 1.5)	0.3	0.01	0.5 (0.2, 0.8)	0.2	0.003	0.0 (− 0.2, 0.3)	0.1	0.84	− 0.1 (− 0.2, 0.0)	0.1	0.07
Sex	− 1.3 (− 3.5, 1.0)	1.1	0.98	4.3 (− 0.2, 8.7)	2.2	0.06	− 2.6 (− 4.7, − 0.4)	1.1	0.02	− 1.6 (− 3.3, 0.2)	0.9	0.08	1.9 (1.2, 2.6)	0.4	< 0.001

*CI* confidence interval, *SE* standard error

## Discussion

Although GH replacement has been used as an effective treatment for idiopathic short stature, whether or not GH therapy can cause progression of scoliosis has remained contentious. We performed this study to investigate the influence of rhGH therapy on radiographic indices of the spine, using propensity-matched analysis. rhGH therapy affected the progression of the scoliosis Cobb angle and apical vertebral translation on the coronal plane. The scoliosis Cobb angle and apical translation increased by 1.0° and 1.2 mm per year, respectively.

Some of the limitations of the study should be addressed before discussing these findings in detail. First, the study was retrospective in nature. In retrospective studies, significant differences are often present between the characteristics of the patient group and the control group. These differences can affect the results of the study. To minimize selection bias, we used propensity-matching analysis. Second, only whole-spine AP radiographs were included in this study. Patient exposure to radiation is an issue of concern for patients and their parents. So, whole-spine AP radiographs were primarily available as they have been preferred as a screening tool for spinal deformities. Scoliosis is a three-dimensional deformity of the spine. We evaluated the progression of the deformity on just the coronal plane of the radiographs, which may have limited the detection of the extent to which rhGH therapy resulted in scoliosis. However, measurement of the scoliosis Cobb angle is the standard method for quantifying spinal curvature [19, 20]. Our results could be meaningful for physicians who treat patients with idiopathic short stature. Further study regarding the three-dimensional deformities of the spine after rhGH therapy will be required. Third, the radiographs taken after the surgery or bracing were excluded from the present study. Therefore, those with severe scoliosis who underwent intervention for scoliosis were not included. Fourth, during adjusting patients’ growth potentials, we used patients’ age rather than the Risser stage, which has been widely used to evaluate growth potentials. Each Risser stage could have a wider period than 1 year. Therefore, we used patients’ age to assess the progression of radiographic indices of the spine in detail, rather than the Risser stage. There could be differences in the progression rate of the radiograph indices according to patients’ age or Risser stage. This issue is beyond the scope of this study and needs to be investigated in a future study. Fifth, there was no consideration of the dose or duration of the GH therapy. Because the doses and durations of rhGH therapy had been changed intermittently according to the patients’ growth status, analysis of the correlation between the dose and duration of rhGH therapy with the progression of scoliosis is hindered. In addition, this study aimed to investigate the influence of rhGH therapy on radiographic indices of the spine. The influence of rhGH therapy protocol on progression of scoliosis is beyond of the scope of this study. We believe that further study will be required because the dose and duration of rhGH therapy may well affect the spinal deformity.

Sex, growth potential, and curve magnitude are factors associated with curve progression in patients with idiopathic scoliosis [21]. A previous study based on the National Cooperative Growth Study reported that rhGH treatment does not appear to increase the risk for scoliosis in children with idiopathic short stature [22]. However, the study [22] focused on the incidence of scoliosis of the patients and did not consider the growth potentials and curve magnitudes of the patients. We considered several factors that might affect the progression of spinal deformity in patients who underwent GH therapy. Radiographic measurements were assessed to see whether patients were affected by GH therapy only after first adjusting for possible confounding factors using propensity score matching and a mixed model. In prospective studies, initial radiographic measurements and patients’ demographics can be controlled. However, the growth rate of the subjects is unpredictable in prospective studies. Because scoliosis progression is influenced by the growth rate [23], the effects of GH therapy on scoliosis can also depend on the growth rate. Therefore, our results are meaningful because age, sex, initial scoliosis Cobb angle, and growth rate, which could affect the progression of spinal deformities, were adjusted to reduce selection bias prior to the analysis.

Whole-spine radiographs have been used for the assessment of spinal deformity as a diagnostic and screening tool. Various measurements can be used to assess spinal deformity. Among them, measurement of the scoliosis Cobb angle is the standard method for quantifying spinal curvature with good overall reliability [14, 19, 20]. Nonetheless, the measurements may vary by 3–10° with the same end-vertebrae [24, 25]. A previous study also reported that the accepted standard for a measured change representing a true change usually has been considered to be 5° [26]. Presently, the annual change of the scoliosis Cobb angle was ~ 1.0°. Progression of the angle may not be found because of measurement error. However, our results showed statistically significant progression of the scoliosis Cobb angle. A patient’s scoliosis Cobb angle can be found to have increased by 10° 10 years later. Therefore, physicians should be aware that regular follow-up is required to evaluate the change in the curvature of the spine in patients with rhGH treatment, even if the patients have not showed progression in the scoliosis Cobb angle for several years.

Little is known of the adverse effects of rhGH in patients with idiopathic short stature [22]. Several studies regarding adverse effects of rhGH on the spine have been performed in patients with genetic disease, such as Turner syndrome [27, 28] and Prader-Willi syndrome [29–31]. In Turner syndrome, patients demonstrate a higher incidence of scoliosis [27]. rhGH therapy does not appear to contribute to an increased incidence but may accelerate progression [12, 32]. The latter results support ours. In Prader-Willi syndrome, the incidence of scoliosis is higher than that of idiopathic scoliosis [29]. However, whether or not rhGH-induced acceleration of linear growth influences the incidence or progression of scoliosis in the patients is uncertain [23]. Although this issue has not been fully addressed, a previous study suggested that increases in paravertebral muscle strength and prolonged muscular asymmetry secondary to GH therapy might be a factor in scoliosis progression [29]. Further study regarding pathophysiology of scoliosis progression after rhGH therapy will be required.

## Conclusions

We investigated the influence of rhGH therapy on radiographic indices of the spine using propensity-matched analysis to control patients’ factors. rhGH therapy affected the progression of the scoliosis Cobb angle and apical vertebral translation on the coronal plane on radiographic examinations. Physicians should be aware that annual follow-up is required to evaluate the changes in the curvature of the spine in patients undergoing rhGH treatment.

## Abbreviations

AP: Anteroposterior
CSVL: Center sacral vertical line
GH: Growth hormone
LMM: Linear mixed model
rhGH: Recombinant human growth hormone
SD: Standard deviation
VC: Variance components
